# Myiasis of the Mastoid Cavity: Case Report

**DOI:** 10.1002/ccr3.71671

**Published:** 2025-12-09

**Authors:** Hannes Hefty, Danilo Esaltato, Georgios Mantokoudis, Sven Beckmann, Samuel Tschopp

**Affiliations:** ^1^ Department of Otorhinolaryngology, Head and Neck Surgery, Inselspital Bern University Hospital and University of Bern Bern Switzerland

**Keywords:** case report, mastoid cavity, myiasis, otology, parasitosis

## Abstract

Mastoid cavity myiasis is a rare but important differential diagnosis. While otomyiasis mainly occurs in tropical regions, it is a diagnosis in patients with a history of radical mastoidectomy presenting with otalgia or otorrhea, particularly in those with diabetes or poor hygiene. Prompt mechanical removal of larvae combined with antiseptic irrigation and systemic antibiotics is an effective and sufficient treatment, typically preventing recurrence or complications.

## Graphical abstract

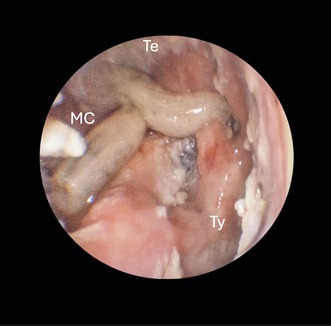

## 1 Introduction

While infections and discharge are common problems in patients with radical mastoidectomy, otomyiasis of the mastoid cavity remains an uncommon condition without standardized treatment. Otomyiasis is defined as a parasitic infestation of the external or middle ear caused by dipterous larvae, with the most commonly observed species belonging to the *Sarcophagidae* and *Calliphoridae* families [1]. While this disease is most prevalent in tropical and subtropical regions—where heat and moisture facilitate the fly's life cycle—cases have been reported worldwide [2, 3, 4, 5]. Other common patient‐related co‐factors include chronic otorrhea, low socioeconomic status, and debilitated individuals [6, 7]. Diagnosis is primarily based on clinical findings and subsequently confirmed by the visualization of larvae during otoscopic or microscopic examination. The cornerstone of treatment involves the physical removal of the larvae, either under local or general anesthesia. Even though topical antibiotic droplets do not directly affect the larvae, they are commonly used to treat secondary infections and soft tissue inflammation [2]. Additionally, antiseptic ear irrigation solutions are also frequently employed [6]. In this case report, we want to emphasize the need for patient management to prevent complications.

## 2 Case History

A 77‐year‐old male was referred to our emergency department with a four‐day history of right‐sided otalgia and a single episode of bloody otorrhea on the same side. Before this episode, the patient was asymptomatic, with an unremarkable otologic history except for a canal wall down mastoidectomy created 42 years ago following a penetrating head trauma. Further comorbidities included cognitive impairment, type 2 diabetes mellitus, and coronary heart disease with pacemaker implantation. In addition, the patient presented in a neglected state, although according to his own statement, he was not homeless.

## 3 Differential Diagnosis and Investigations

**FIGURE 1:**
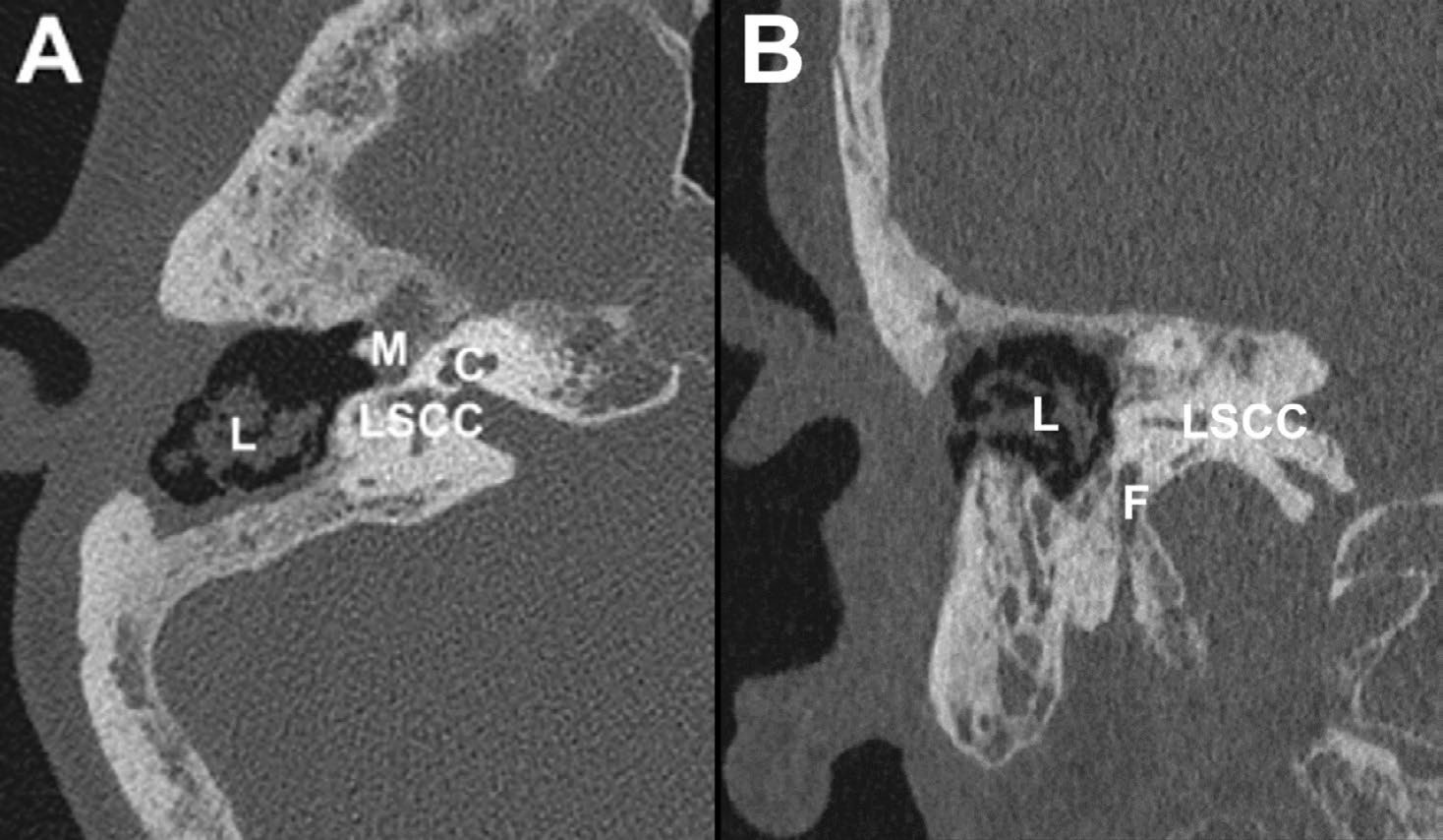
Computed tomography of the temporal bone in axial (A) and coronal (B) planes showing multiple soft‐tissue densities compatible with larvae occupying the radical mastoid cavity. Normal anatomical landmarks are indicated: C, cochlea; F, facial nerve; L, larvae; LSCC, lateral semicircular canal; M, malleus.

**FIGURE 2:**
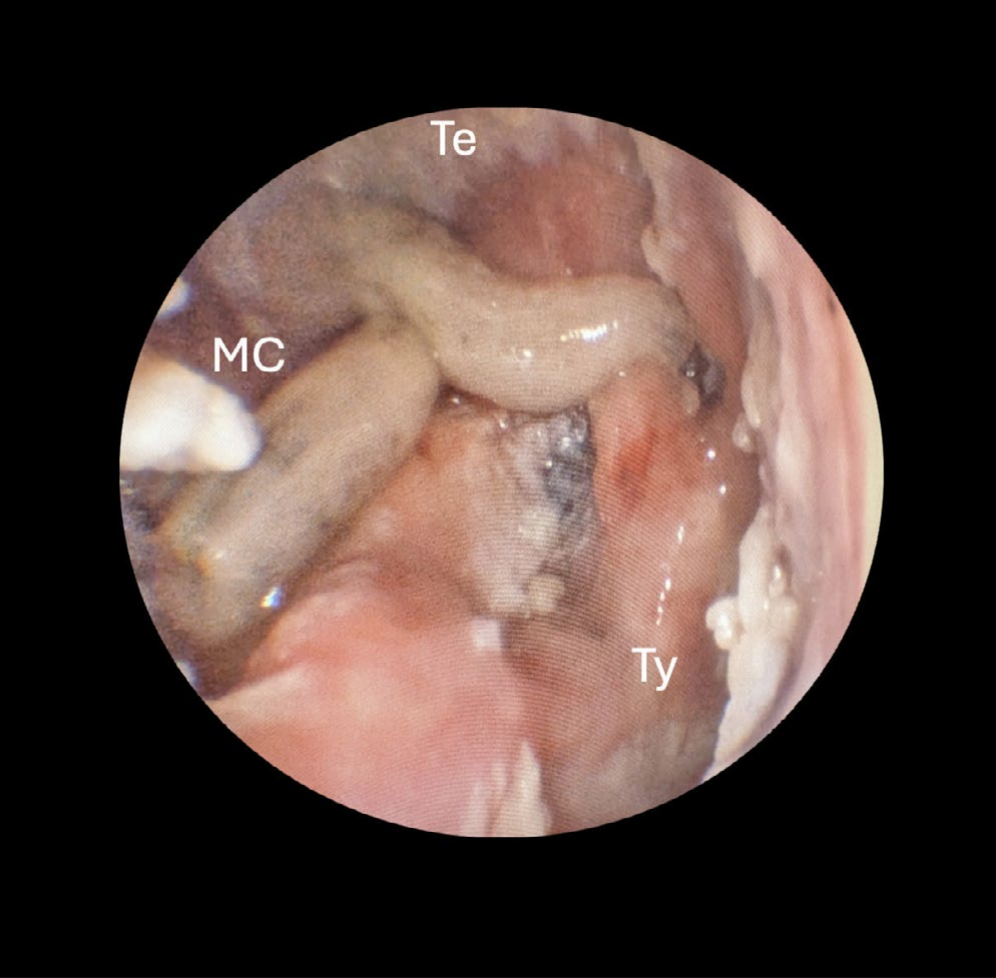
Otoendoscopic view of the canal wall down mastoidectomy cavity after partial clearance of debris and larvae. The anatomical structures are indicated for orientation: MC, mastoid cavity; Te, tegmen tympani; Ty, tympanic membrane.

A computed tomography (CT) scan ordered by the referring physician revealed partial obstruction of the radical cavity with soft tissue without any evidence of complications (Figure 1). On physical examination, multiple larvae were observed moving within the right external auditory canal and radical cavity (Figure 2). The tympanic membrane was intact with inflammatory alterations. A video in the Supporting Information shows an endoscopic view of the living larvae (Video S1). The Weber test lateralized to the right ear; the Rinne test was negative on the right side. No facial nerve dysfunction was noted.

## 4 Treatment and Follow‐Up

**FIGURE 3:**
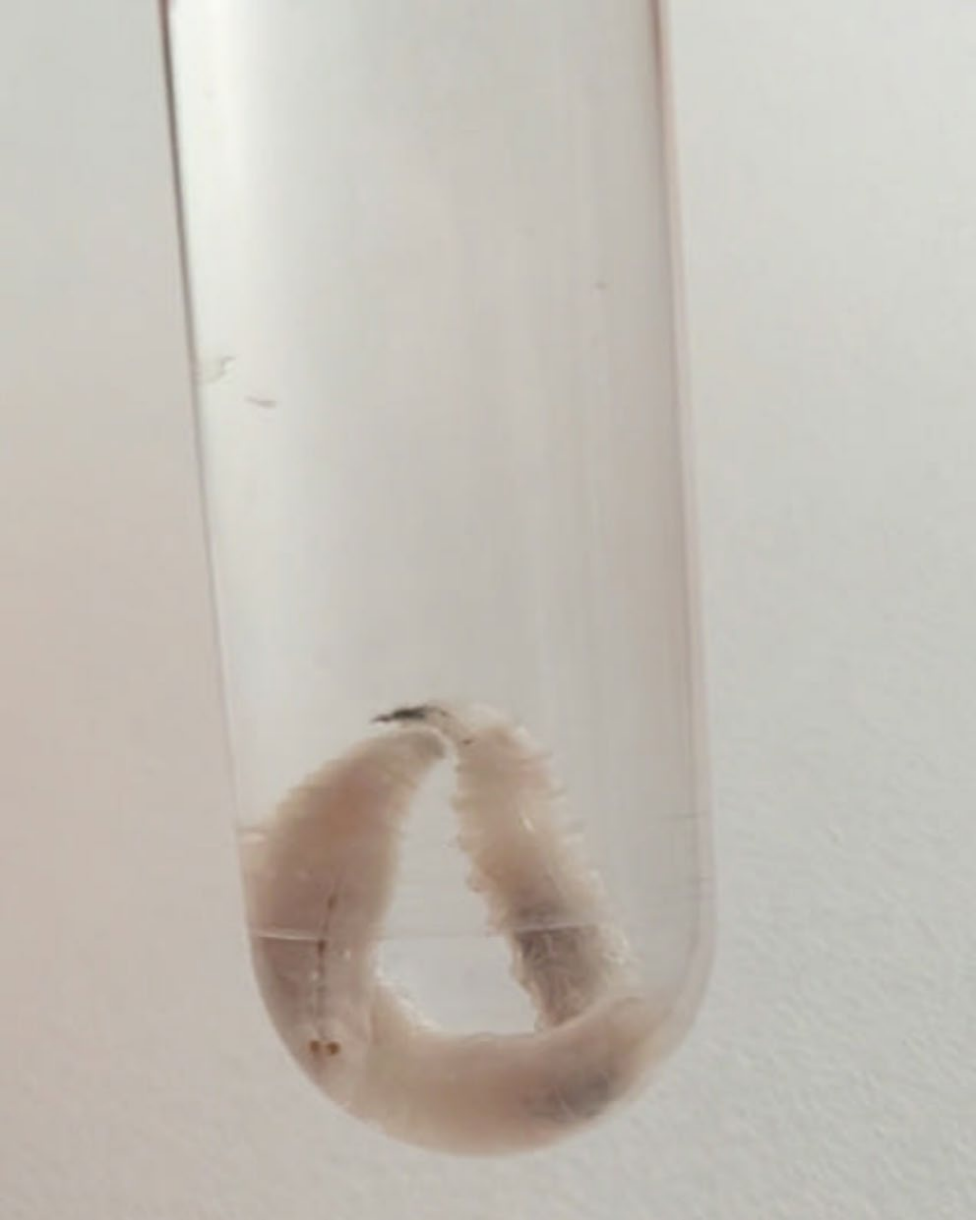
Two specimens of larvae extracted from the mastoid cavity during otomicroscopic examination and debridement.

Treatment consisted of mechanical removal of the larvae (Figure 3), which was performed without the need for local anesthesia. Around 20 larvae were removed and sent to the infectious disease department for identification. Unfortunately, the exact species could not be identified. Following consultation with the infectious disease department, a 14‐day course of systemic Amoxicillin‐Clavulanate and systemic Ciprofloxacin was started to treat the secondary bacterial infection. Additionally, he underwent daily in‐office ear irrigation with a diluted (1:10) povidine‐iodine solution with 0.9% sodium chloride. Microscopic examination revealed fly larvae as pathogenic organisms, without further entomological specification. Subsequent scheduled follow‐up evaluations at 2 and 6 weeks post‐treatment were uneventful, with no recurrence of larvae or signs of infection.

## 5 Discussion

While multiple reports of myiasis in the ear exist [3, 4, 5, 7], mastoid cavity myiasis is an exceptionally rare condition, with only three cases reported in the literature to date [2]. The earliest documented case was reported back in 1949 by Brauneck, who first treated a 27‐year‐old patient with myiasis in the mastoid cavity after radical mastoid surgery for chronic otitis media. This patient was managed with mechanical removal of larvae and saline irrigation [8]. In 2004, Uzun et al. described a second case involving a 31‐year‐old patient with myiasis in the radical cavity that occurred 10 years after a gunshot wound and radical mastoidectomy. The treatment involved mechanical removal of larvae and topical irrigation with H_2_O_2_ several times [9]. The last case was reported in the United States, involving a 55‐year‐old homeless patient who underwent creation of a radical cavity to control chronic ear infections, with CT imaging findings consistent with a canal wall‐down mastoidectomy [2]. This patient was managed with a combination of mechanical larval extraction, surgical debridement, and antibiotic‐steroid ear drops. Previous reports of otomyiasis are summarized in Table 1.

**TABLE 1:** Summary of published case reports describing patients with myiasis of the mastoid cavity, including patient characteristics, treatment, and clinical outcome.

Article	Year, country	Patient characteristics	Treatment	Outcome
Brauneck [8]	1949, England	27‐year old prisoner of war	Mechanical removal of 57 larvae and saline irrigation	Resolution
Uzun et al. [9]	2004, Turkey	31‐year old patient	Ether‐soaked cotton pack followed by mechanical removal, topical application of 4% boric acid with alcohol	Resolution
Kahane et al. [2]	2019, USA	55‐year‐old homeless patient	Mechanical removal of 30 larvae followed by operative debridement and topical antibiotic‐steroid drop	Resolution

Otomyiasis is a condition that mainly occurs in tropical and subtropical regions. However, these cases from England [8], Turkey [9], and the USA [2] illustrate that the disease also occurs in countries of the global north. Myiasis occurs when a female fly deposits eggs or first instar larvae in the ear canal, leading to larval infestation. While the exact criteria for infection remain unclear, frequent cofactors include poor sanitary conditions, chronic otitis media, previous otological surgery, poor socioeconomic status, alcoholism, and diabetes mellitus [1]. In our case, a history of radical mastoidectomy, diabetes mellitus, and poor living conditions was present as contributing factors.

Although indications for canal wall down mastoidectomies have decreased in recent years, elderly, diabetic, and debilitated individuals who have previously undergone this procedure appear to remain particularly vulnerable to myiasis of the radical cavity. Due to the rarity of mastoid cavity otomyiasis, no standardized guidelines exist for its diagnosis and management. Diagnosis is primarily clinical, based on the visualization of larvae within the cavity. We suggest assessing the radical cavity primarily by endoscopy, with computed tomography scan reserved to rule out complications such as bony erosion. While myiasis‐related meningitis is a theoretical concern, no cases have been reported associated with otomyiasis [1]. One possible reason is that *Hypodermatinae* species, primarily responsible for the latter condition, have not been observed in otomyiasis cases, highlighting the importance of larval identification [1, 6].

**FIGURE 4:**
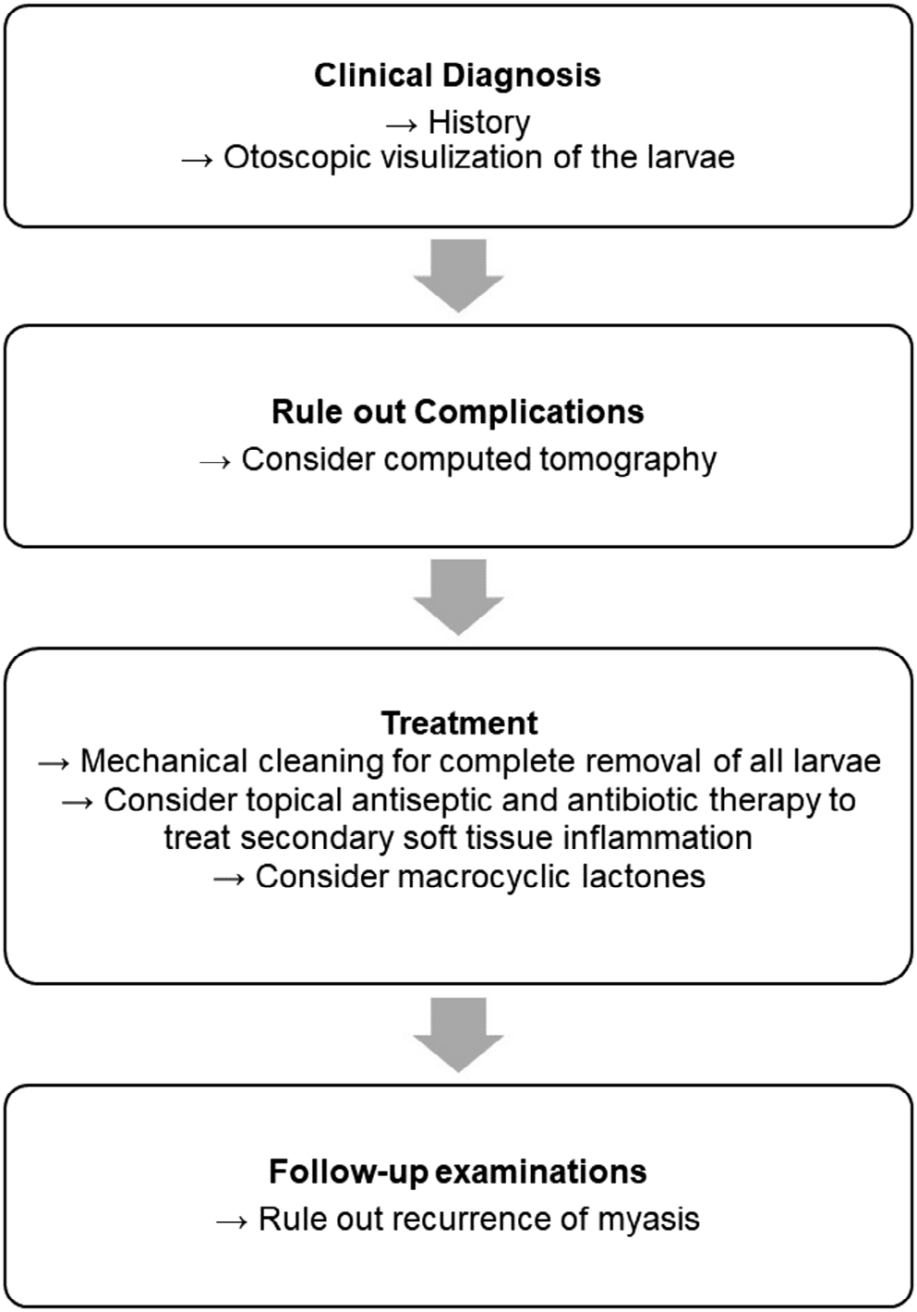
Overview of the proposed diagnostic workup and management strategies for otomyiasis, including myiasis of the mastoid cavity.

Treatment strategies for the radical cavity myiasis should align with those used in standard otomyiasis cases. General recommendations are summarized in Figure 4. The mainstay of treatment comprises the mechanical removal of larvae, which was performed in all cases. Additionally, topical therapy with antibiotic or antiseptic ear drops is typically used to address secondary soft‐tissue inflammation. Alternatively, rinsing with saline or macrocyclic lactones may be viable options.

Finally, three out of four patients recovered without needing a surgical intervention. Thus, we reserve surgical therapy for extensive cases and cases in which thorough cleaning of the radical cavity cannot be achieved under local anesthesia.

## 6 Conclusion

Mastoid cavity otomyiasis is an extremely rare condition most likely favored by poor hygiene. Treatment with mechanical removal of larvae, regular irrigation, systemic antibiotics, and antibiotic and antiseptic droplets is usually sufficient. Surgical debridement might be necessary in selected cases.

## Author Contributions

**Hannes Hefty:** conceptualization, data curation, visualization, writing – original draft, writing – review and editing. **Danilo Esaltato:** conceptualization, data curation, visualization, writing – review and editing. **Georgios Mantokoudis:** conceptualization, project administration, supervision, visualization, writing – review and editing. **Sven Beckmann:** conceptualization, data curation, project administration, supervision, visualization, writing – review and editing. **Samuel Tschopp:** conceptualization, data curation, methodology, project administration, supervision, visualization, writing – original draft, writing – review and editing.

## Funding

The authors have nothing to report.

## Consent

Written informed consent was obtained from the patient to publish this case report and any accompanying images or data. All efforts were made to ensure patient anonymity.

## Conflicts of Interest

The authors declare no conflicts of interest.

## Supporting information


**Video S1:** Video showing the live larvae within the mastoid cavity.

## Data Availability

The data from this case report is available from the corresponding author upon reasonable request, Samuel Tschopp.

## References

[1] M. Rodríguez‐Ruiz , A. Acosta , E. Cifuentes‐Cardozo , M. Chirveches , and D. Rosselli , “Otomyiasis: Systematic Review,” International Archives of Otorhinolaryngology 23 (2019): 104–109, 10.1055/s-0037-1617427. 30647793 PMC6331295

[2] J. Kahane , C. Longworth , C. Dickson , and M. Ng , “Mastoid Cavity Myiasis: A Case Report and Review of the Literature,” Otology & Neurotology 40 (2019): e627–e630, 10.1097/MAO.0000000000002261. 31045903

[3] A. Koirala , T. N. Yogi , S. Dhamel , et al., “Aural Myiasis in a Healthy Adult Laborer From Nepal: A Case Report,” Annals of Medicine and Surgery 87 (2025): 3889–3893, 10.1097/MS9.0000000000003281. 40486618 PMC12140780

[4] M. Tbini , H. Jaafoura , M. Ghabi , E. Chebil , and M. Bensalah , “Otomyiasis Caused by *Musca domestica* in a Child: A Case Report,” International Journal of Surgery Case Reports 94 (2022): 107108, 10.1016/j.ijscr.2022.107108. 35468383 PMC9046598

[5] P. Feka‐Homsy , A. G. L'Huillier , L. Monod , E. Monin , N. Guinand , and J.‐M. Schwob , “ *Cochliomyia hominivorax* Aural Myiasis in a 7‐Year‐Old Traveler,” IDCases 41 (2025): e02327, 10.1016/j.idcr.2025.e02327. 40735452 PMC12305604

[6] J. Jervis‐Bardy , N. Fitzpatrick , A. Masood , G. Crossland , and H. Patel , “Myiasis of the Ear: A Review With Entomological Aspects for the Otolaryngologist,” Annals of Otology, Rhinology, and Laryngology 124 (2015): 345–350, 10.1177/0003489414557021. 25358614

[7] A. Barlaam , L. Putignani , S. Pane , P. M. Bianchi , R. A. Papini , and A. Giangaspero , “What's in a Child's Ear? A Case of Otomyiasis by *Sarcophaga argyrostoma* (Diptera, Sarcophagidae),” Parasitology International 87 (2022): 102537, 10.1016/j.parint.2022.102537. 34995772

[8] H. W. F. Brauneck , “A Case of Human Myiasis,” BMJ 2 (1949): 1335, 10.1136/bmj.2.4640.1335. 15396849 PMC2052093

[9] L. Uzun , F. Cinar , L. B. Beder , T. Aslan , and K. Altintas , “Radical Mastoidectomy Cavity Myiasis Caused by *Wohlfahrtia magnifica* ,” Journal of Laryngology and Otology 118 (2004): 54–56, 10.1258/002221504322731655. 14979975

