# Supplementary figures and images for: Myiasis of the Mastoid Cavity: Case Report
Source: Clin Case Rep. 2025 Dec 9;13(12):e71671. doi: 10.1002/ccr3.71671 (PMC12689269; doi:10.1002/ccr3.71671)

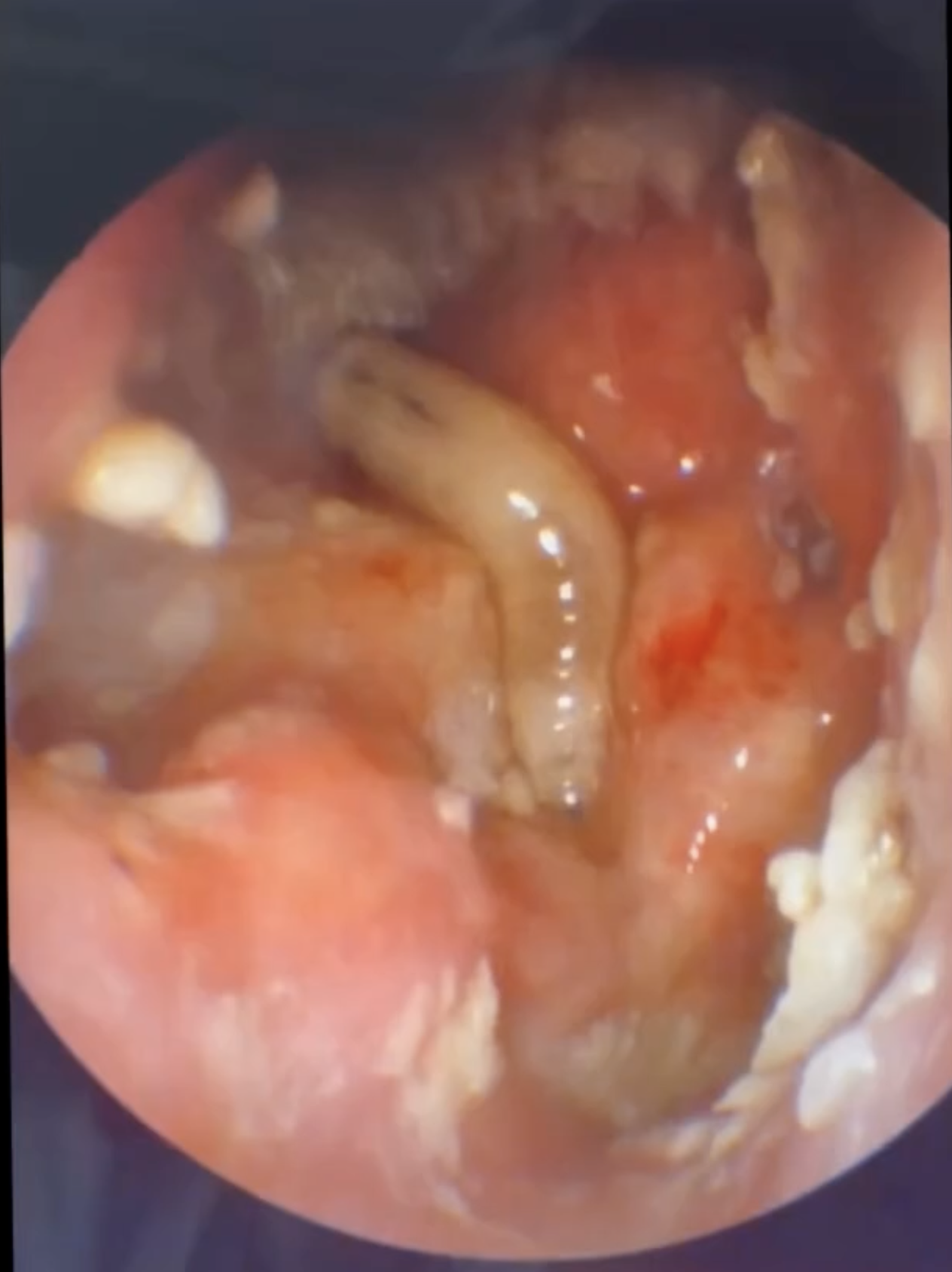

Supplement: Supplementary file 1 — Video S1: Video showing the live larvae within the mastoid cavity. [file CCR3-13-e71671-s001.zip › Place Holder Supplemental Video.png]
